# 20 years on – the measurement of blood pressure and detection of hypertension in children and adolescents: a national descriptive survey

**DOI:** 10.1038/s41371-023-00846-6

**Published:** 2023-07-15

**Authors:** Lily Jones, Julie Park, Joanne Blair, Daniel B. Hawcutt, Gregory Y. H. Lip, Alena Shantsila

**Affiliations:** 1 Department of Women’s and Children’s Health, University of Liverpool, Liverpool, United Kingdom; 2 Department of Endocrinology, Alder Hey Children’s NHS Foundation Trust, Liverpool, United Kingdom; 3 NIHR Alder Hey Clinical Research Facility, Alder Hey Children’s NHS Foundation Trust, Liverpool, United Kingdom; 4 Liverpool Centre for Cardiovascular Science at University of Liverpool, Liverpool John Moores University and Liverpool Heart and Chest Hospital, Liverpool, United Kingdom

**Keywords:** Health care, Risk factors

## Abstract

In 1997 a survey identified a general lack of standardisation of blood pressure (BP) measurement and little consensus on the criteria for diagnosing hypertension amongst paediatricians. We have conducted a new online survey in 2021, to compare clinical practice between the two time periods. A national quality improvement survey was approved by the GAPRUKI committee and then circulated to consultant-grade general paediatricians. 125 analysable replies from 34 different sites were received and compared with the 1997 data. 106 (84.8%) reported clinic nurse involvement in BP measurement, more than twice than reported previously (40.6%). Most paediatricians (53.6%) now rely on oscillometric devices, whereas the mercury sphygmomanometer was favoured previously (82.7%). If assessing BP manually (*n* = 89), most (79.8%) now use Korotkoff phase V as the auscultatory endpoint for diastolic BP (phase IV was previously used (52.1%)). Diagnostic criteria of hypertension, the criteria (≥95^th^ centile for gender, age and height) were constant, and 100% of paediatricians diagnosed it using systolic BP, but only 43 (34.4%) used diastolic BP, a decrease from 79.4% previously. Ambulatory BP Monitoring was six times more available than in 1997 (81.6% vs 13.6%). Similar to previous findings, only 12 (9.6%) paediatricians would manage hypertensive patients themselves, however 82 (72.6%) would keep general paediatric input. There have been important changes in the assessment of BP in children, including increased nurse involvement and greater use of technology. However, fewer paediatricians are responding to high diastolic pressures than twenty years ago.

## Introduction

**Fig. 1:**
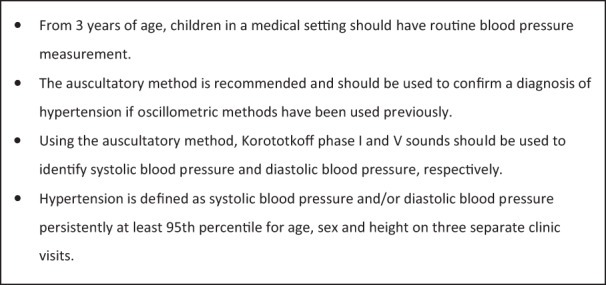
Key recommendations for blood pressure measurement and management in the paediatric population.

A previous study performed just over twenty years ago, suggested a lack of standardisation of blood pressure (BP) measurement techniques and little consensus on the criteria for diagnosing hypertension amongst consultant-grade paediatricians in the United Kingdom (UK) and Ireland [1]. Updated clinical practice guidelines on the diagnosis and management of hypertension in paediatric patients have since been published (Fig. 1) [2–6]. Although promoting routine BP measurement similarly, these guidelines demonstrate inconsistencies in the categorisation of BP status [7, 8], and the introduction of fixed cut-offs at 13-years of age in the 2017 American Academy of Paediatrics guidance has been particularly controversial [9].

The implementation of evidence-based guidelines into daily practice is a recognised challenge [10]. Unsurprisingly, adherence to paediatric hypertension guidelines in a recent multi-centre study, including a variety of communities across the United States, was sub-optimal [11]. To our knowledge, there is no available data from the UK and Ireland.

For the facilitation of BP measurement in paediatric clinics, there should be availability of a wide selection of cuff sizes to allow for large variation in upper arm size. The measurement of BP in the paediatric population, especially in restless young or anxious children, can be technically challenging [12]. These environmental and human factors, combined with the absence of a clear definition constituting abnormal BP values, can make measurement and interpretation of BP more difficult in paediatric compared to adult patients.

To further investigate current clinical practice and interpretation of BP measurement and treatment of hypertension in children and adolescents in the United Kingdom and Ireland, we conducted an online survey for constulant-grade General Paediatricians. This was distributed via the General and Adolescent Paediatric Research in the United Kingdom & Ireland (GAPRUKI) network, an organisation established in June 2016 to facilitate multi-centre paediatric research. This survey was consistent with the survey sent twenty years previously, to enable evaluation of how clinical practice has changed between the two time periods [1]. Our aim is to describe the approach of consultant-grade General Paediatricians to the measurement and interpretation of BP in children and adolescents in 2021 as compared to the 1997 survey.

## Methods

Modelled on the postal survey from twenty years ago [1], a Microsoft Form was devised and sent to the GAPRUKI committee for review. Following agreed revisions, the survey link was sent via email to all GAPRUKI mailing list members in November 2021, with an accompanying body of text outlining the rationale behind the study (Supplementary Material 1). Members of GAPRUKI were invited to participate and distribute the survey to their consultant-grade General Paediatric colleagues. Reminder emails were sent to all GAPRUKI mailing list members, regardless of response, due to the anonymity of the questionnaire, a week after initial posting and a week before the survey deadline. Participation in the study was voluntary. It was advertised to take approximately 5–10 min to complete and consisted of 17 required questions, and dependant on selected responses, a maximum of four further optional follow-up questions. The survey was live for 8 weeks in total. All data were automatically entered into Microsoft Excel for analysis. The survey was anonymous, however, to ensure that our data was representative of practice across the UK and Ireland, participants were required to disclose their NHS Trust employer. Ethical approval was not required for this Quality Improvement survey.

In keeping with the descriptive nature of the study, participant answers from the 2021 survey will be subjectively compared to the 1997 survey. In the 1997 survey, not all respondents answered every question, and the percentages reported reflect the number of responses received for each individual question. Although designed using the survey from twenty years ago [1], modifications before general mailing meant that not all questions had identical response options.

## Results

At the time of mailing there were 103 GAPRUKI members, who were affiliated with 53 different NHS Hospital Trusts. We received survey responses from 23 of these Trusts (43% Trust response rate). The responses from 11 additional NHS Trusts were received from the survey being sent onto colleagues by GAPRUKI members, giving a total of 34 different NHS Hospital Trusts. The number of respondents per Trust varied from one to ten.

Two participants, who were identified by participant disclosure as either not consultant-grade general paediatricians or currently working in the UK or Ireland, were excluded, leaving 127 respondents. Of these, two respondents said that BP measurement was not relevant to their clinical practice, therefore there were 125 analysable replies. The absolute number of responses was 683 in the 1997 survey [1]. On average, the questionnaire took participants 07:05 min to complete.

### Blood pressure measurement

In the 2021 survey, 114 (91%) responded that the circumstances behind routinely measuring BP was dependant entirely on the clinical presentation. Of these respondents, 110 (97%) would measure at any age, including from birth, if clinically indicated. Compared to the 1997 data, more paediatricians reported that they would measure BP at any age (Table 1). Of the 456 (68.6%) respondents to this question in 1997, 256 (56.1%) would not routinely measure BP in children below the age of three years (Table 1).

**Table 1:** Age of BP screening, patient posture and identity of measurer in paediatric practice.

	2021 survey, *n* (%)	1997 survey, *n* (%)
(i) Age when routine BP measurement performed in an outpatient		
From birth	13 (10)	118 (17.7)
From 1 years	0 (0)	82 (12.3)
From 3 years	2 (2)	153 (20.0)
From 7 years	1 (1)	80 (12.0)
From 13 years	0 (0)	23 (3.5)
Never	N/A	209 (31.4)
Other	3 (2)	N/A
At any age if clinically indicated	106 (85)	N/A
(ii) Posture for BP measurement in outpatient clinic		
Seated	87 (70)	407 (60.0)
Supine	8 (6)	124 (18.3)
No preference to position	20 (16)	147 (21.7)
Both seated and supine in the same patient	10 (8)	N/A
(iii) Diastolic end point measurement in outpatient clinic		
Phase 4 (muffling of sounds)	16 (13)	348 (51.0)
Phase 5 (disappearance)	71 (57)	213 (31.2)
Both phase 4 and 5	2 (2)	106 (15.5)
No reply / Not applicable	36 (29)	16 (2.3)
(iv) Who measures BP		
Paediatrician	19 (15)	358 (59.4)
Clinic nurse	62 (50)	54 (9.0)
Either	44 (35)	190 (31.6)

*BP* Blood Pressure, *N/A* Not Applicable.

In the clinic, only 19 (15%) paediatricians reported consistently recording BP themselves, whilst 44 (35%) reported measurements were taken by either themselves or a nurse colleague (Table 1). Clinic nurse involvement in BP measurement was reported by 106 (85%) paediatricians, a percentage more than double the rate of nursing involvement reported in 1997 (Table 1). The chosen posture for BP measurement was reported as seated in 87 (70%), supine in eight (6%), and 20 (16%) had no preference (Table 1). There were 10 (8%) respondents who preferred to routinely measure both seated and supine BP in the same patient. These data are consistent with the data from 1997 (Table 1).

One paediatrician reported no access to BP cuffs in clinic, whilst most (96%) reported access to at least three different cuff sizes (Table 2). These results are similar to those reported in 1997 (93.0%) (Table 2). In 2021, most paediatricians (54%) relied on automatic or semi-automatic BP oscillometric devices, 24 (19%) used an aneroid sphygmomanometer and 2 (2%) a mercury sphygmomanometer. All three types were used interchangeably by 32 (26%) paediatricians (Table 2). This greatly differed from 1997 data, where the mercury sphygmomanometer was used most frequently (82.7%) (Table 2). For further investigation of suspected hypertension, 102 (82%) of paediatricians had access to Ambulatory BP Monitoring (ABPM). ABPM was reported to be six times more available than in 1997 (Table 2).

**Table 2:** Equipment available for BP measurement in children.

	2021 survey, *n* (%)	1997 survey, *n* (%)
(i) Number of BP cuff sizes available		
0	1 (1)	N/A
1	0 (0)	8 (1.4)
2	4 (3)	32 (5.6)
3	39 (31)	343 (32.5)
4 or more	81 (65)	343 (60.5)
(ii) Type of sphygmomanometer used		
Mercury sphygmomanometer	2 (2)	565 (82.7)
Aneroid	24 (19)	36 (5.3)
Automatic or semi-automatic oscillometric device.	67 (54)	64 (9.4)
All types	32 (26)	18 (2.6)
(iii) Ambulatory BP monitoring available		
Yes	102 (82)	93 (13.6)

*BP* Blood Pressure, *N/A* Not Applicable

### Diastolic end-points

If the paediatricians were measuring BP manually (*n* = 89), the most favoured (79.8%) auscultatory end-point for measuring diastolic BP was the disappearance of sounds (Korotkoff phase V) (Table 1). This differed from the 1997 data, where majority used the muffling of sounds (Korotkoff phase IV), although there was considerable variation in these data (Table 1).

### The clinical diagnosis of hypertension

All paediatricians used systolic BP for a diagnosis of hypertension: 26 (21%) would not take diastolic readings into account, 56 (45%) required both systolic and diastolic pressures to be raised and 43 (34%) responded to either raised systolic or diastolic pressures (Table 3). No paediatricians reported to respond to a raised diastolic pressure alone, which contrasts from the 465 (79.4%) who reported to do so in the 1997 data (Table 3).

**Table 3:** The clinical diagnosis and management of hypertension in children.

	2021 survey, *n* (%)	1997 survey, *n* (%)
(i) Reporting hypertension		
Systolic alone	26 (21)	105 (17.9)
Diastolic alone	0 (0)	79 (13.5)
Both systolic and diastolic	56 (45)	16 (2.7)
Either systolic or diastolic	43 (34)	386 (65.9)
(ii) BP centile to diagnose hypertension		
90^th^	12 (10)	76 (12.9)
95^th^	85 (68)	246 (41.8)
99^th^	23 (18)	N/A
Other	5 (4)	N/A
>95^th^	N/A	267 (45.3)
(iii) Measuring leg blood pressure in a hypertensive child		
Yes, routinely	17 (14)	204 (30.3)
No	24 (19)	173 (25.7)
Yes, if clinically indicated	84 (67)	296 (44.0)
(iv) Measuring blood pressure in both arms in a hypertensive child		
Yes, routinely	49 (39)	299 (43.9)
No	8 (6)	225 (33.0)
Yes, if clinically indicated	68 (54)	157 (23.1)
(v) Medical management		
Manage	12 (10)	76 (11.4)
Refer	113 (90)	591 (88.6)

*BP* Blood Pressure, *N/A* Not Applicable.

Readings at the 95^th^ percentile in relation to gender, age and height were used to diagnose hypertension by 85 (68%) of paediatricians (Table 3). Pressures at the 90^th^ percentile were used by 12 (10%) paediatricians and 23 (18%) used the 99^th^ percentile for diagnosis (Table 3). In the 1997 survey, 513 (86.3%) paediatricians made a diagnosis based on measurements at or greater than the 95^th^ percentile (Table 3).

Most paediatricians (55%) relied on raised serial BP measurements over a morning, afternoon, or day for a diagnosis of hypertension. Of the paediatricians who responded with visit numbers (*n* = 56), 45 (80%) would require at least three visits where BP was raised before treating or referring their patient for management of hypertension.

### The management of the hypertensive child

Routinely measuring BP in the legs of a hypertensive child was reported by 17 (14%) paediatricians, and a further 84 (67%) reported that they would do so if clinically indicated (Table 3). In contrast, 49 (39%) paediatricians reported that they routinely measure BP in both arms and 68 (54%) would do so if clinically indicated in a child with hypertension (Table 3). In a hypertensive child, fewer paediatricians reported never measuring BP in both arms (6%) compared to the legs (19%), which differed to responses in 1997 (Table 3).

Only 12 (10%) paediatricians, of which six (50%) had a specialist interest in either paediatric cardiology or nephrology, would manage these patients themselves. The remainder of paediatricians would refer to specialists, although 82 (73%) would continue to see the child in the general paediatric clinic. Nephrology was the preferred specialty for referall for 65 (58%) paediatricians. This was consistent with findings from 1997 (Table 3).

## Discussion

These data demonstrate that some progress has been made in improving availability and use of equipment to measure BP in the past two decades. For the diagnosis of hypertension, the present survey responses suggest a lower threshold than previously, however paediatricians are much less likely to respond to high diastolic readings.

Expert guidelines recommendations influence clinician’s attitudes and clinical practice. The two main guidelines for BP measurement and management in children and adolescents are the European Society of Hypertension (ESH) and the American Academy of Paediatrics (AAP) guidelines, which were recently updated in 2016 and 2017, respectively [2, 3].

Our survey shows that for routine BP measurement, most respondents answered in concordance with current guideline recommendations and will measure BP at any age if clinically indicated. Both guidelines recommend routine BP measurement from the age of three years, and only in younger children where risk factors for HTN are present [2, 3]. In otherwise healthy individuals, the ESH guideline recommends biennial BP measurement [3], whereas the AAP guideline recommends annual measurement [2].

The majority of the survey responders would measure BP in the seated position, using an oscillometric device, as per both guidelines recommendations. It has to be noted that the device model needs to be validated for the paediatric population [2, 3]. The almost complete disappearance of mercury sphygmomanometer use in clinical practice is a direct result of safety concerns arising in the early 2000’s [13]. In keeping with this knowledge and technological advances of the past two decades, when compared to the 1997 data, wider availability of ABPM for the investigation of hypertension, and more common routine use of automatic or semi-automatic BP oscillometric devices were reported [1]. Other changes reflective of general changes in medical care over the past twenty years were demonstrated, such as the increased prevalence of nurse involvement in clinic BP measurement.

For a diagnosis of hypertension, both guidelines still recommend auscultatory-confirmed measurement [2, 3]. It is somewhat concerning that 28.8% of respondents from the present survey reported that measuring of diastolic end-point was not applicable to their clinical practice, as this could suggest that they are not confirming BP measurement via auscultatory methods. Alternatively, paediatricians clinical practice may reflect knowledge of the limited precision of oscillometric device diastolic BP measurement relative to systolic BP measurement [14, 15]. Of those respondants who specified a favoured diastolic end-point, the majority (79.4%) used Korotkoff phase V, which is endorsed by both guidelines [2, 3]. At the time of mailing of the 1997 survey, recommendations were changing and there was considerable confusion on the correct auscultatory end-point for the measurement of diastolic BP [16–18], which was reflected in the survey responses [1].

Both guidelines recommend defining hypertension as a systolic or diastolic BP of at least 95^th^ percentile for age, gender and height measured clinically on three separate occasions [2, 3]. The data from 1997 suggests closer adherence to these recommendations [1]. The present survey responses suggests paediatricians have a lower threshold for the diagnosis of hypertension than previously, however are much less likely to respond to high diastolic readings. The clinical significance of this change in practice deserves further consideration. Most general paediatricians in both the present and 1997 survey would refer their hypertensive patients to the appropriate specialists, which is also recommended by both guidelines [1–3]..

The present survey is likely to have encountered similar limitations of the postal survey sent twenty years previously [1]. As with all self-reported clinical practice surveys, the possibility of reported practice differing from actual practice cannot be discounted. Furthermore, the option of “when clinically indicated” was heavily favoured when included as a possible response in survey questions. This is somewhat problematic, as it gives no indication of the clinical circumstances that inform decision making, and it is likely that these differ between paediatricians. The possibility that the clinical practice of the responders differed from the non-responders also cannot be excluded. The survey was sent via the GAPRUKI network, so responders were likely to have an interest in paediatric and adolescent research, leading to over-representation of research-workers among our respondents. The clinical practice of clinicians in the North West of England was also over-represented, although there was at least one respondent employed by every regional team in NHS England and respondents from Scotland, Wales and Northern Ireland.

Our questionnaire was delivered on Microsoft Forms, an online platform that clinicians should be familiar with, and efforts were made to make the questionnaire clear and concise. Despite these factors, and our two reminder emails, we received considerably fewer analysable replies compared to the postal questionnaire twenty years previously [1]. A likely contributing factor is practitioner fatigue from correspondence received by email and various survey/feedback invitations. Personal letter by post could have resulted at a higher response yield. Despite this, we feel our sample size is suitable to assess the usual current clinical practice of consultant-grade general paediatricians.

The GAPRUKI network differs from the BPA, who distributed the 1997 survey, in being a network for general paediatricians only. The 1997 survey may have received responses from sub-specialist consultant paediatricians, such as paediatric nephrologists, who may have a particular view on BP measurement and management, which should be appreciated when comparing the responses.

From comparing responses from the present questionnaire to those collected twenty years previously, it is evident that BP measurement using auscultatory methods are more standardised now, however paediatricians are more likely to rely on oscillometric technology. It is important to note that paediatric BP reference data were derived from data collected from manual readings, and these might not be directly applicable to measurements made using oscillometric technology. There is greater availability of BP equipment and technology, yet disparity remains in the classification of paediatric hypertension and fewer paediatricians are responding to high diastolic pressures than twenty years ago. It is reassuring that appropriate specialist input is sought when required. However, an increase in hypertension and a decline in the cardiovascular health of the paediatric population might be anticipated in line with the high rates of childhood obesity, which may put extra demands on these specialist services. Overall, in the past two decades some progress has been made in improving availability and use of equipment to measure BP, for detection of hypertension in children and adolescents.

## Summary

### What is known about topic

- In a suvey done twenty years ago blood pressure measurement and the diagnosis of hypertension in children and adolescents lacked standardisation.

### What this study adds

- Clinical practice has changed and there is greater use of technology and more clinic nurse involvement in blood pressure measurement.
- Disparity in the classification of paediatric hypertension remains and fewer paediatricians are responding to high diastolic measurments than 20 years ago.
- This study contributes to raising awareness of need of blood pressure measurement in children and adolescents.

### Supplementary information

Supplemental

## Data Availability

Original data are available from the corresponding author on reasonable request.
