# Supplementary material for: 20 years on – the measurement of blood pressure and detection of hypertension in children and adolescents: a national descriptive survey
Source: J Hum Hypertens. 2023 Jul 15;37(12):1086–90. doi: 10.1038/s41371-023-00846-6 (PMC10739225; doi:10.1038/s41371-023-00846-6)
Supplement: Supplementary file 1 — Supplemental [file 41371_2023_846_MOESM1_ESM.pdf]

20 Years on - The measurement of BP and detection of hypertension in CAYP

20 Years ago, data from a similar questionnaire suggested a general lack of standardisation of BP measurement techniques and little consensus on the criteria for diagnosing hypertension amongst paediatricians. (1) Updated guidelines on hypertension in paediatric patients have since been published. We think that it would be extremely interesting and important to assess if these guidelines have resulted in changes to clinical practice. Our findings will help to evaluate whether progress has been made and identify further ways to standardise and improve patient care.

As a consultant-grade paediatrician, we would be very grateful if you would take the time to complete our questionnaire.
Time to complete = Approximately 5 -10 minutes

(1) - Lip GY, Beevers M, Beevers DG, Dillon MJ. The measurement of blood pressure and the detection of hypertension in children and adolescents. J Hum Hypertens. 2001 Jun;15(6):419-23. PubMed PMID: 11439318. Epub 2001/07/06. eng.

1. What is your job role? *

- ◯ Consultant
- ◯ Other

2. Please state your role.

3. Please state your specialist interest if applicable.

4. Please state the NHS Trust that you are employed by. *

5. Do you routinely measure BP in outpatient's clinic? *

- ◯ At first appointment
- ◯ At each appointment
- ◯ Depends on clinical question
- ◯ No – Not relevant to clinical practice

6. At what age do you start measuring routine BP in clinic? *

- ◯ Birth
- ◯ 1 Year
- ◯ 3 Years
- ◯ 7 Years
- ◯ 13 Years
- ◯ Other
- ◯ Measure at any age if clinically indicated

7. What number of BP cuff sizes are available to you in your clinic? *

- ◯ 0
- ◯ 1
- ◯ 2
- ◯ 3
- ◯ 4 or more

8. Who would usually measure BP in clinic? *

- ◯ Doctor
- ◯ Nurse
- ◯ Either

9. What posture for BP measurement do you prefer in clinic? *

- ◯ Seated
- ◯ Supine
- ◯ No preference to position
- ◯ Both seated and supine in the same patient

10. What type of sphygmomanometer is used? *

- ◯ Mercury
- ◯ Aneuroid (no liquid)
- ◯ Automatic or semi-automatic
- ◯ All types

11. If measuring BP manually, how do you report diastolic BP? *

- Korotkoff phase IV (muffling)
- Korotkoff phase V (disappearance)
- Report both
- Not applicable

12. How do you report hypertension? *

- Systolic alone
- Diastolic alone
- Both systolic and diastolic
- Either systolic or diastolic

13. Which BP percentile do you use to diagnose hypertension? *

- 90th
- 95th
- 99th
- Other

14. Which percentile charts do you use as a reference for "normal parameters"?

15. On how many occasions would a child need to be hypertensive for you to treat or refer? *

- 1
- 2
- 3
- 4 or more
- After serial measurements over a morning/afternoon/day

16. If you wanted to further evaluate a child with presumed hypertension, would you have access to Ambulatory BP monitoring? *

- Yes
- No

17. As an inpatient which limb would you use to routinely assess BP? *

- Arm
- Leg

18. Would you ever measure leg BP in a hypertensive child? *

- Yes, routinely
- No
- Only if clinically indicated

19. Would you ever measure BP in both arms in a hypertensive child? *

- Yes, routinely
- No
- Only if clinically indicated

20. Would you manage hypertension yourself? *

- Manage myself
- Refer to specialist

21. Please state which department you would refer to.

22. If you referred the child to a specialist, would you continue seeing the child yourself?

- Yes
- No

This content is neither created nor endorsed by Microsoft. The data you submit will be sent to the form owner.

Microsoft Forms
